# Penicillin and cephalosporin cross-reactivity: role of side chain and synthetic cefadroxil epitopes

**DOI:** 10.1186/s13601-020-00368-1

**Published:** 2020-12-04

**Authors:** Gador Bogas, Cristobalina Mayorga, Ángela Martín-Serrano, Rubén Fernández-Santamaría, Isabel M. Jiménez-Sánchez, Adriana Ariza, Esther Barrionuevo, Teresa Posadas, María Salas, Tahía Diana Fernández, María José Torres, María Isabel Montañez

**Affiliations:** 1 Allergy Research Group, Instituto de Investigación Biomédica de Málaga-IBIMA, Hospital Civil, 29009 Málaga, Spain; 2 Allergy Unit, Hospital Regional Universitario de Málaga, Hospital Civil, 29009 Málaga, Spain; 3 Nanostructures for Diagnosing and Treatment of Allergic Diseases Laboratory, Andalusian Center for Nanomedicine and Biotechnology-BIONAND, Parque Tecnológico de Andalucía, 29590 Málaga, Spain; 4 Departamento de Biología Celular, Genética y Fisiología, Universidad de Málaga, 29071 Málaga, Spain; 5 Departamento de Medicina, Universidad de Málaga, Facultad de Medicina, 29071 Málaga, Spain

**Keywords:** Amoxicillin, Betalactam, Cephalosporin, Cross-reactivity, Drug allergy, Antigenic determinant, Specific IgE

## Abstract

**Background:**

Analysis of cross-reactivity is necessary for prescribing safe cephalosporins for penicillin allergic patients. Amoxicillin (AX) is the betalactam most often involved in immediate hypersensitivity reactions (IHRs), and cefadroxil (CX) the most likely cephalosporin to cross-react with AX, since they share the same R1 side chain, unlike cefuroxime (CO), with a structurally different R1. We aimed to analyse cross-reactivity with CX and CO in patients with confirmed IHRs to AX, including sIgE recognition to AX, CX, CO, and novel synthetic determinants of CX.

**Methods:**

Fifty-four patients with confirmed IHRs to AX based on skin test (ST) and/or drug provocation test (DPT) were included. Serum sIgE to AX and benzylpenicillin was determined by Radioallergosorbent test (RAST). Two potential determinants of CX, involving intact or modified R1 structure, with open betalactam ring, were synthesised and sIgE evaluated by RAST inhibition assay.

**Results:**

Tolerance to CX (Group A) was observed in 64.8% cases and cross-reactivity in 35.2% cases (Group B). Cross-reactivity with CO was only found in 1.8% cases from Group B. ST to CX showed a negative predictive value of 94.6%. RAST inhibition assays showed higher recognition to CX as well as to both synthetic determinants (66% of positive cases) in Group B.

**Conclusions:**

Cross-reactivity with CX in AX allergic patients is 35%, being ST not enough for prediction. R1, although critical for recognition, is not the unique factor. The synthetic determinants of CX, **1**-(HOPhG-Ser-Bu) and **2**-(pyrazinone) are promising tools for determining in vitro cross-reactivity to CX in AX allergic patients.

## Background

**Fig. 1:**
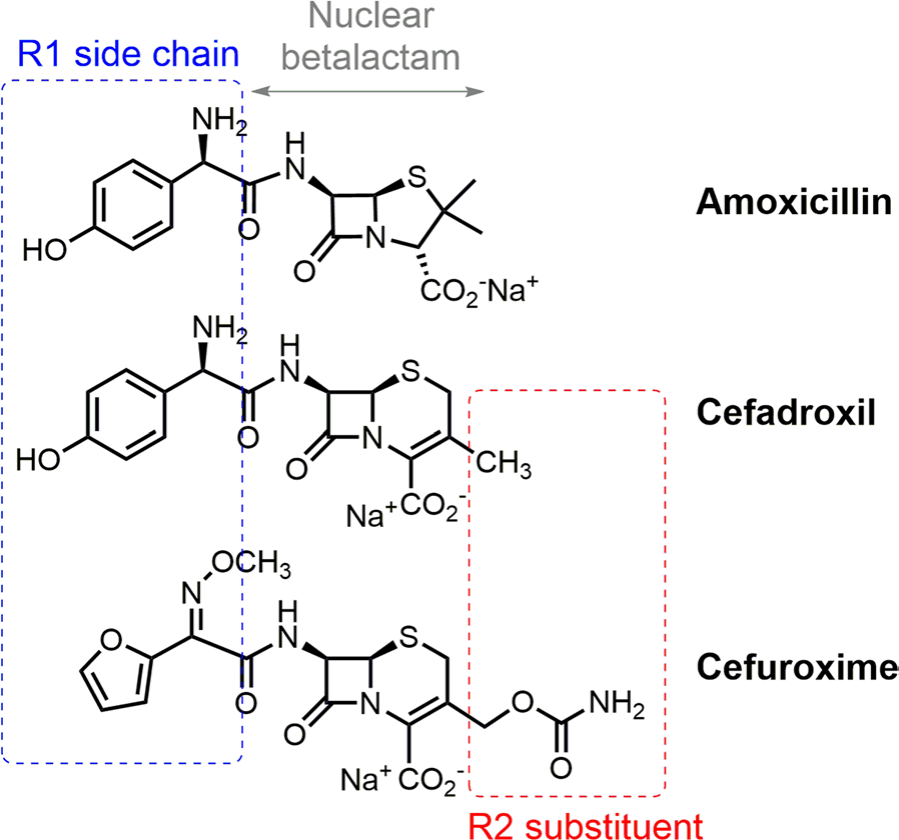
Chemical structures of betalactam antibiotics involved in the study: amoxicillin (AX), cefadroxil (CX), and cefuroxime (CO); with indication of the different parts of the structures

Betalactams (BLs) are the drugs most frequently involved in immediate (IgE-mediated) hypersensitivity reactions (IHRs) [1–3], which could be explained by their ability to act as haptens due to their high chemical reactivity against proteins [4, 5]. BL chemical structure is formed by a 4-membered ring (the so-called BL ring) that in penicillins is fused to a 5-membered thiazolidine ring, and in cephalosporins to a 6-membered dihydrothiazine ring (Fig. 1). These drugs have a side chain (R1) bound to the BL ring; besides, cephalosporins have a second side chain (R2) bound to the dihydrothiazine ring, whose chemical structures distinguish the different compounds [6, 7].

Penicillins are the most consumed antibiotics in Europe, representing 37% of total consumption, followed by cephalosporins with a 15% of total antibiotic consumption [8]. Among them, amoxicillin (AX) is the most consumed and the most often involved in IHRs to BLs followed by cephalosporins [3, 9] which include the following: cefuroxime (CO), ceftriaxone, cefatrizin, cefaclor, and cefadroxil (CX) [10, 11], with different percentage of cross-reactivity between them [6], highly related to their chemical structure [12–14]. Cross-reactivity rate with cephalosporins in penicillin-allergic patients with IgE-mediated reactions ranges from 0% to almost 40% depending on the chemical structure of the BL involved [15–22], specifically on similarity in the R1 side chain [23, 24]. In this context, AX, which shares the same amino R1 side chain with CX (Fig. 1), could have a high cross reactivity [19–21]. Conversely, CO, with a different R1 side chain, has shown tolerance in patients with IHRs to penicillins [16–19] and, more recently, similar results have been found with cefazolin and ceftibuten [22, 25].

Cross-reactivity has important clinical implications, especially for searching safe alternative for further treatments, and an accurate diagnosis based on skin testing (ST) is recommended, being the role of drug provocation tests (DPT) controversial [3, 9, 26]. In vitro evaluation of cross-reactivity to BLs, mainly based on immunoassays, is limited by the difficulty for studying the structure of cephalosporin-protein conjugates [27]. Although several reports have addressed this issue [28–30], the antigenic determinants of cephalosporins are currently not well-known [31].

To our knowledge, structure–activity relationship (SAR) studies have been the unique successful approach for investigating cephalosporin epitopes [28–30, 32]. In this context, we have elucidated precise epitope structures through synthesis and immunologic evaluation of well-defined structures proposed as antigenic determinants for cephalosporins with different R1, bearing different functionalities at the C-6 of the cephalosporin (methyl, hydroxymethyl, aldehyde, mercaptomethyl) and without involvement of the remaining dihydrothiazine ring [29, 30]. Moreover, we have identified a novel synthetic pyrazinone structure as an antigenic determinant of cefaclor [28], formed after reaction of the amino group in the R1 with the likely aldehyde functionality at C-6 of the original cephalosporin [28, 32]. CX is another aminocephalosporin that could follow the same fragmentation and reactivity pathways as cefaclor [32].

In this study we have evaluated the in vivo degree of cross-reactivity with CX and CO in patients with confirmed IHR to AX and the immunological recognition of AX and these cephalosporins by serum specific IgE (sIgE). The ultimate aim of this study was to evaluate if synthetic structures, proposed as potential antigenic determinants mimicking the fragment of CX, which would remain coupled to the protein, can help get insight into the structure responsible for CX allergies and, therefore, study cross-reactivities between AX and CX.

## Methods

### Patients

The studied group was obtained from the Regional University Hospital of Málaga Drug Allergy Database. This prospective cohort includes all patients with confirmed drug allergy from 1984 to 2019 after an allergological workup including clinical history, ST, and DPT.

**Fig. 2:**
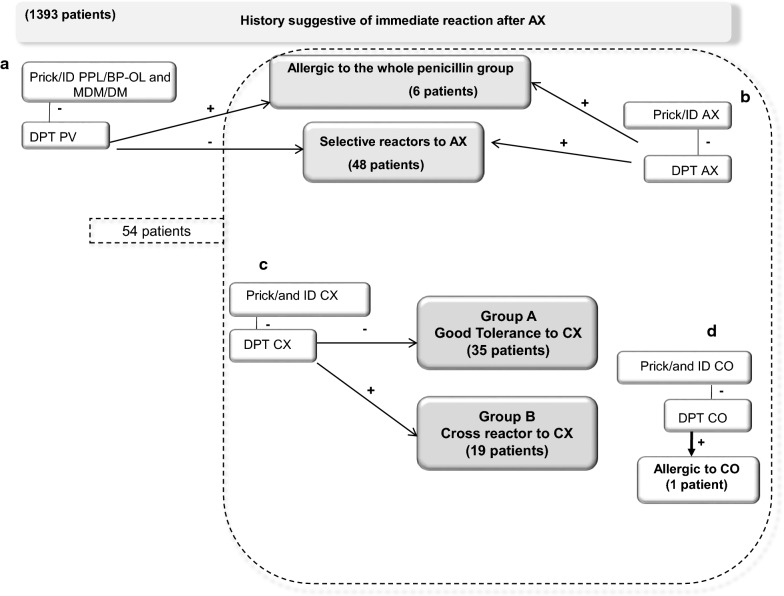
**a** The diagnostic algorithm includes skin tests (STs) to PPL/BP-OL, MDM/DM and amoxicillin (AX) and if negative drug provocation tests (DPT) to penicillin V (PV) and AX. Patients were classified into two groups, allergic to the whole group of penicillins or selective reactors to AX. **b** Cross reactivity to cefadroxil (CX) was analysed by STs and DPT and AX-allergic patients classified into two groups: Group A with good tolerance and Group B with cross-reactivity. In all cases cross-reactivity with cefuroxime (CO) was also analysed by ST and DPT

Patients with IHR to AX (allergic to the whole penicillin group or AX selective reactors with good tolerance to penicillin V (PV)) were diagnosed following the European Academy of Allergy and Clinical Immunology (EAACI) recommendations [9, 33]. Tolerance to CX and CO was evaluated and, based on CX tolerance, patients were classified into: Group A with tolerance (demonstrated by negative ST and DPT) and Group B with cross-reactivity (demonstrated by positive ST or DPT) (Fig. 2).

### Skin test

Skin prick (SPT) and, if negative, intradermal tests (IDT) were performed as described [9, 33], using benzylpenicilloyl-poly-L-lysine (PPL, DAP, Diater, Leganés, Spain) at 1.07·10^–2^ M, minor determinant mixture (MDM: benzylpenicillin, benzylpenicilloate, and benzylpenilloate) at 1.5 M and AX (Diater laboratories, Madrid, Spain); CX (Lilly SA, Madrid) and CO (GlaxoSmithKline S.A, Madrid) all at 20 mg/mL. Since May 2011 DAP composition has changed and includes the major determinant benzylpenicilloyl-octa-L-lysine (BP-OL) at 0.04 mg/mL, equivalent to 8.64·10^–5^ M concentration of the benzylpenicilloyl (BPO) moiety, and the minor determinant (MD) at 0.5 mg/mL, equivalent to 1.5·10^–3^ M concentration of sodium benzylpenilloate. Cephalosporin reagents were prepared according to Romano [19, 34].

Readings were done after 20 min and considered positive: (i) In SPT, if a wheal larger than 3 mm surrounded by erythema appeared, with a negative response to the control saline; (ii) In IDT, if the increase in diameter of the wheal area marked initially was greater than 3 mm surrounded by erythema. Positive data expressed as the mean diameter recorded by measuring the largest and the smallest diameters at right angles to each other [35].

### Drug provocation test

In subjects with negative ST to PPL/BP-OL and MDM/MD, oral DPT with PV was performed at incremental dose (50, 100, 100, 150 mg) each 40-min until reaching the total cumulative dose (TCD) of 400 mg, followed by a 2 day therapeutic course of PV of 400 mg/8-h at home [33]. If DPT with PV and ST to AX was negative, oral DPT with AX was performed (50, 100, 150, 200 mg) until TCD of 500 mg, followed by a 2 day therapeutic course of AX 500 mg/8-h at home. For cross-reactivity analysis, if ST was negative, CX was orally administered (50, 100, 150, 200 mg) until TCD of 500 mg, followed by a 2 day therapeutic course of CX 500 mg/8-h. Finally, CO was administered following this procedure.

Patients were carefully monitored during DPT and for 2 h after the last dose, complete equipment for cardiopulmonary resuscitation was available [36].

### In vitro sIgE determination by radioallergosorbent test (RAST)

It was done using BP and AX conjugated to Poly-L-Lysine (PLL) (Sigma, St. Louis, MO) resulting in BPO-PLL and AXO-PLL in the solid phase, as described [37, 38], and radiolabeled anti-IgE antibody (kindly provided by Thermo Fisher Scientific and radiolabelled in our laboratory) [28]. Samples were considered positive if they were higher than 2.5% of label uptake, which was the mean + 2SD of a negative control group.

### Synthesis of chemical structures

**Fig. 3:**
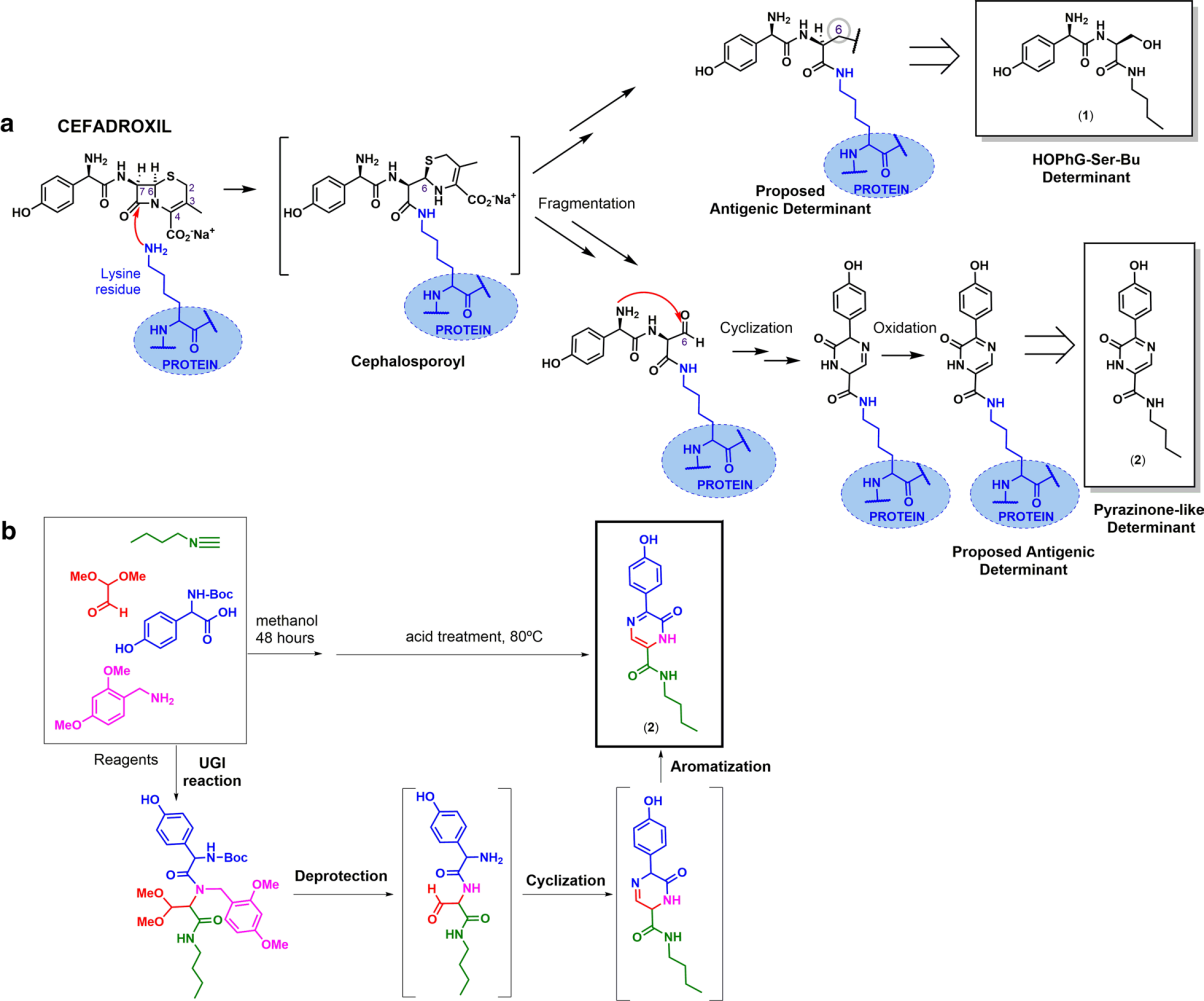
**a** Degradation hypothesis of cefadroxil (CX) after nucleophilic opening of betalactam ring by protein amino groups, leading to the cephalosporoyl intermediate, which degrades through dihydrotiazine fragmentation, and leading different functionality at carbon 6, hydroxyl and aldehyde respectively, and eventually resulting in the proposed antigenic determinants. Those equivalent synthetic structures for further immunological evaluation are represented in the square. **b** Synthesis of pyrazinone (molecule 2), pyrazin-2(1H)-one, proposed as CX determinant, through Ugi/Deprotect/Cyclize strategy

The molecule **1** (HOPhG-Ser-Bu) (Fig. 3a) was synthesised as described [30].

The molecule **2** (pyrazinone) (Fig. 3a) was synthesised following the Ugi/Desprotect/Cyclize strategy (Fig. 3b) [39], adapting protocols from cefaclor pyrazinone synthesis [28]. The synthetic methodology and characterisation of the pure compound can be found in this article's Additional file 1.

### RAST inhibition assay

This was done as described [38], incubating sera from patients with RAST values higher than 7% with different BLs (AX, CX, and CO) and the synthetic determinants of CX (**1** and **2**) in two ten-fold decreasing concentrations (100 mM and 10 mM) for 18 h at room temperature. After this, the AXO-PLL disc was added, followed by the previous described RAST procedure. The results were expressed as percentage inhibition with respect to the non-inhibited serum. Comparison of the inhibition capacity of the different inhibitors was made at 50% inhibition.

### Statistical analysis

Description of quantitative variable included the median, mean, standard deviation (SD), and interquartile range (IR). Differences in percentage between the groups were compared by Chi-square analysis, numeric demographic data by Student t test. Comparisons for variables without a normal distribution were performed by the Mann–Whitney test for non-related samples and by Friedman test for related samples. All statistical analyses were done using the software package GraphPad PRISM v7. A P < 0.05 was considered statistically significant.

## Results

From 1393 patients with confirmed BL hypersensitivity evaluated from 1984 to 2019, 994 subjects were confirmed with IHRS to AX, from which we randomly selected 54 patients, whose cross-reactivity to CX and CO was evaluated and flow-charts analysed (Fig. 2). The mean age was 41.7 ± 12.04 years; 35 (64.8%) were males; 51 (94.4%) had 1 episode and 3 (5,6%) two; in 32 (56.1%) episodes AX-CLV was the culprit and in 25 (43.9%) AX. The main symptoms were anaphylaxis in 40 (70.2%) cases, urticaria in 13 (22.8%), and anaphylactic shock in 4 (7%). The mean time interval between drug administration and development of symptoms was 26.1 ± 19.2 min and between last reaction and study 132.4 ± 131.4 days (Table 1).

**Table 1:** Demographic and clinical data of patients included in the study

Pat	Group	Sex	Age	Drug	Epi	Reaction	IDR (min)	IRS (d)
1	A	M	55	AX-CLV	1	Anaphylaxis	60	180
2	B	M	44	AX-CLV	1	Anaphylaxis	20	60
3	A	M	46	AX	1	Anaphylaxis	30	30
4	B	M	55	AX	1	Urticaria	30	300
5	A	M	62	AX-CLV	1	Anaphylaxis	60	90
6	B	F	45	AX-CLV	1	Anaphylaxis	10	30
7	B	M	24	AX	1	Anaphylaxis	10	30
8	B	F	45	AX	1	Anaphylaxis	20	90
9	A	M	47	AX	2	Urticaria Anaphylaxis	40 10	477 365
10	A	F	46	AX-CLV	1	Anaphylaxis	30	120
11	A	M	43	AX-CLV	1	Anaphylaxis	45	60
12	B	F	40	AX/AX-CLV	2	Urticaria Anaphylaxis	30 5	112 109
13	A	F	16	AX-CLV	1	Urticaria	60	30
14	A	M	27	AX-CLV	1	Anaphylactic shock	5	21
15	A	M	66	AX	1	Anaphylaxis	60	365
16	B	M	44	AX	1	Anaphylaxis	10	365
17	A	F	50	AX-CLV	1	Anaphylaxis	15	30
18	A	M	44	AX-CLV	1	Anaphylaxis	10	280
19	A	M	25	AX-CLV	1	Urticaria	60	120
20	B	F	33	AX-CLV	1	Anaphylaxis	15	6
21	B	F	36	AX-CLV	1	Anaphylaxis	30	60
22	A	M	30	AX	1	Anaphylaxis	5	20
23	B	M	45	AX-CLV	1	Anaphylactic shock	5	30
24	A	M	30	AX-CLV	1	Anaphylaxis	30	210
25	B	M	57	AX-CLV	1	Anaphylaxis	5	60
26	A	M	49	AX	1	Anaphylaxis	10	120
27	A	F	39	AX	1	Urticaria	40	40
28	A	F	39	AX-CLV	1	Urticaria	60	95
29	A	F	21	AX-CLV	1	Anaphylaxis	15	230
30	A	M	46	AX-CLV	1	Anaphylaxis	2	137
31	B	M	49	AX	1	Anaphylaxis	5	180
32	B	F	37	AX-CLV	1	Anaphylaxis	20	30
33	A	F	57	AX	1	Anaphylaxis	30	90
34	B	M	26	AX	1	Anaphylaxis	20	10
35	B	M	48	AX-CLV	1	Anaphylaxis	60	146
36	A	M	23	AX-CLV	1	Anaphylaxis	50	60
37	B	F	42	AX	1	Anaphylaxis	5	120
38	B	M	57	AX-CLV	1	Anaphylaxis	30	28
39	B	M	30	AX-CLV	1	Urticaria	10	730
40	A	F	37	AX-CLV	1	Anaphylaxis	5	30
41	A	F	14	AX-CLV	1	Anaphylaxis	10	90
42	A	M	44	AX	1	Urticaria	30	120
43	A	M	28	AX	2	Anaphylaxis Anaphylaxis	20 25	230 180
44	A	M	63	AX-CLV	1	Anaphylaxis	10	30
45	A	M	51	AX	1	Urticaria	60	30
46	A	M	35	AX	1	Urticaria	30	90
47	A	M	32	AX	1	Anaphylaxis	45	180
48	A	M	53	AX	1	Anaphylaxis	30	90
49	A	M	44	AX	1	Urticaria	45	120
50	A	F	38	AX-CLV	1	Anaphylaxis	10	30
51	A	M	54	AX-CLV	1	Anaphylactic shock	5	90
52	A	F	62	AX-CLV	1	Urticaria	60	210
53	B	M	49	AX-CLV	1	Anaphylaxis	30	120
54	A	F	44	AX	1	Anaphylactic shock	5	240

Patients were classified into Group A (Good tolerance to cefadroxil) or Group B (Cross-reactivity with cefadroxil)

Pat, Patients; M, Male; F, Female; AX-CLV, Amoxicillin-clavulanic; AX, Amoxicillin; Epi, Number of episodes; IDR (min), Interval drug administration and development of symptoms in minutes; IRS (d), Interval last reaction and allergological study in days

### Allergological work-up

Fifty (92.6%) patients were diagnosed by ST and 4 (7.4%) by DPT (Tables 2 and 3). Regarding ST, 2 (4%) cases were positive to PPL/BP-OL (both by IDT), 4 (8%) to MDM/MD (all by IDT), and to AX 27 (54%) by SPT and 23 (46%) by IDT (Table 2). Moreover, P37 and P39 developed immediate generalised pruritus and wheals 20 and 30 min respectively after ST with AX.

**Table 2:** Skin tests and RAST results in patients from Group A (Good tolerance to cefadroxil) and Group B (Cross-reactivity with cefadroxil)

Pat	Group	Skin test					RAST	AX-PLL
		PPL/BP-OL	MDM/MD	AX	CX	CO	BPO-PLL	
1	A	Neg	Neg	Neg	Neg	Neg	ND	ND
2	B	Neg	Neg	SPT + (5 x 5)	ID + (2x2)	Neg	0	3.42
3	A	Neg	Neg	SPT+(5 x 5)	Neg	Neg	0.15	3.24
4	B	Neg	Neg	SPT+(4 x 5)	SPT + (4 x 4)	Neg	0	0
5	A	Neg	Neg	Neg	Neg	Neg	0	0
6	B	Neg	Neg	SPT + (5 x 5)	IDT + (1 x 1)	Neg	ND	ND
7	B	Neg	Neg	SPT + (6 x 6)	SPT + (3 x 3)	Neg	0.34	14.59
8	B	Neg	Neg	SPT + (8 x 1)	SPT + (5 x 5)	Neg	0	3.51
9	A	Neg	Neg	SPT + (5 x 5)	Neg	Neg	0	0
10	A	Neg	Neg	IDT + (3 x 2)	Neg	Neg	0	0
11	A	Neg	Neg	IDT + (2 x 2)	Neg	Neg	0	0
12	B	Neg	Neg	IDT + (3 x 3)	IDT + (2x2)	Neg	0.2	0.46
13	A	Neg	Neg	IDT + (4 x 4)	Neg	Neg	ND	ND
14	A	Neg	Neg	SPT + (3 x 3)	Neg	Neg	0	0
15	A	Neg	Neg	IDT + (2 x 3)	Neg	Neg	ND	ND
16	B	Neg	Neg	SPT + (4 x 5)	IDT + (2 x 2)	Neg	1.72	11.71
17	A	Neg	Neg	IDT + (2 x 3)	Neg	Neg	0	0
18	A	Neg	Neg	IDT + (3 x 4)	Neg	Neg	0	0
19	A	Neg	Neg	Neg	Neg	Neg	ND	ND
20	B	Neg	Neg	IDT + (2 x 2)	IDT + (2 x 1)	Neg	0	3.2
21	B	Neg	Neg	SPT + (5 x 6)	IDT + (1 x 2)	Neg	ND	ND
22	A	IDT+(2x1)	IDT+(1x1)	SPT + (3 x 3)	Neg	Neg	3.2	6.79
23	B	Neg	IDT+(2x2)	SPT + (6 x 6)	SPT + (3 x 3)	Neg	23.54	29.83
24	A	Neg	Neg	SPT + (2 x 3)	Neg	Neg	0	3.03
25	B	Neg	Neg	SPT + (5 x 6)	IDT + (2 x 2)	Neg	1.22	7.55
26	A	Neg	Neg	Neg	Neg	Neg	0	0
27	A	Neg	Neg	IDT + (3 x 3)	IDT + (2 x 2)	Neg	0.2	0.46
28	A	Neg	Neg	IDT + (3 x 4)	Neg	Neg	2.15	8.32
29	A	Neg	Neg	IDT + (4 x 4)	Neg	Neg	0.09	0.54
30	A	Neg	Neg	SPT + (5 x 6)	Neg	Neg	0	1.14
31	B	Neg	Neg	SPT + (5 x 5)	SPT+(2+3)	Neg	0	22.54
32	B	Neg	Neg	SPT + (3 x 3)	IDT+(2 x 3)	Neg	0	15.56
33	A	Neg	Neg	SPT + (4 x 5)	Neg	Neg	1.28	2.31
34	B	Neg	Neg	SPT + (6 x 6)	IDT + (2 x 3)	Neg	1.87	3.36
35	B	Neg	IDT + (2x2)	SPT + (7 x 8)	SPT + (3 x 4)	Neg	6.15	26.09
36	A	Neg	Neg	IDT + (2 x 2)	Neg	Neg	0	25.56
37	B	Neg	Neg	IDT + (1 x 2)	Neg	Neg	0.37	0.13
38	B	Neg	Neg	SPT + (5 x 6)	IDT + (3 x 4)	Neg	1.65	21.8
39	B	Neg	Neg	IDT + (2 x 2)	Neg	Neg	0	0.92
40	A	Neg	Neg	SPT + (2 x 3)	Neg	Neg	0	0.7
41	A	Neg	Neg	IDT + (5 x 6)	Neg	Neg	0	1.93
42	A	Neg	Neg	IDT + (2 x 2)	Neg	Neg	0	0.37
43	A	Neg	Neg	IDT + (3 x 3)	Neg	Neg	0	1.24
44	A	Neg	Neg	IDT + (4 x 5)	Neg	Neg	0.23	0
45	A	Neg	Neg	IDT + (2 x 2)	Neg	Neg	0	23.2
46	A	Neg	Neg	SPT + (4 x 4)	Neg	Neg	0	0
47	A	Neg	ID + (2x2)	SPT + (5 x 6)	Neg	Neg	0.84	1.12
48	A	ID + (2x2)	Neg	SPT + (4 x 5)	Neg	Neg	8.13	13.87
49	A	Neg	Neg	IDT + (2 x 2)	Neg	Neg	0	0.14
50	A	Neg	Neg	SPT + (5 x 4)	Neg	Neg	0.54	0.41
51	A	Neg	Neg	IDT + (5 x 7)	Neg	Neg	0	14.68
52	A	Neg	Neg	IDT + (3 x 4)	Neg	Neg	0	7.93
53	B	Neg	Neg	SPT + (4 x 5)	Neg	Neg	0	16.84
54	A	Neg	Neg	IDT + (3 x 2)	Neg	Neg	0.06	33.02

Pat, Patients; PPL/BPO-OL, Benzylpenicilloyl-poly-L-lysine/benzylpenicilloyl-octa-L-lysine; MDM/MD, Minor determinant mixture/minor determinant; AX, Amoxicillin; CX, Cefadroxil; CO, Cefuroxime; BPO-PLL, Benzylpenicilloyl-poly-L-lysine; AXO-PLL, Amoxicilloyl-poly-L-lysine; SPT, Skin prick test; IDT, Intradermal test; Neg, Negative; ND, Not done

**Table 3:** Drug provocation test results in patients from Group A (Good tolerance to cefadroxil) and Group B (Cross-reactivity with cefadroxil)

Pat	Group	Drug	Reaction	IDR (min)	TCD (mg)
1	A	AX	Urticaria	160	500
		PV/CX/CO	Good tolerance	–	–
2	B	PV/CO	Good tolerance	–	–
3	A	PV/CX/CO	Good tolerance	–	–
4	B	PV/CO	Good tolerance	–	–
5	A	AX	Anaphylaxis	15	50
		PV/CX/CO	Good tolerance	–	–
6	B	PV/CO	Good tolerance	–	–
7	B	PV/CO	Good tolerance	–	–
8	B	PV	Anaphylaxis	45	150
		CO	Good tolerance	–	–
9	A	PV/CX/CO	Good tolerance	–	–
10	A	PV/CX/CO	Good tolerance	–	–
11	A	PV/CX/CO	Good tolerance	–	–
12	B	PV/CO	Good tolerance	–	–
13	A	PV/CX/CO	Good tolerance	–	–
14	A	PV/CX/CO	Good tolerance	–	–
15	A	PV/CX/CO	Good tolerance	–	–
16	B	PV/CO	Good tolerance	–	–
17	A	PV/CX/CO	Good tolerance	–	–
18	A	PV/CX/CO	Good tolerance	–	–
19	A	AX	Urticaria	140	500
		PV/CX/CO	Good tolerance	–	–
20	B	PV/CO	Good tolerance	–	–
21	B	PV/CO	Good tolerance	–	–
22	A	CX/CO	Good tolerance	–	–
23	B	CO	Good tolerance	–	–
24	A	PV/CX/CO	Good tolerance	–	–
25	B	PV/CO	Good tolerance	–	–
26	A	AX	Generalized pruritus and erythema	60	150
		PV/CX/CO	Good tolerance	–	–
27	A	PV/CO	Good tolerance	–	–
28	A	PV/CX/CO	Good tolerance	–	–
29	A	PV/CX/CO	Good tolerance	–	–
30	A	PV/CX/CO	Good tolerance	–	–
31	B	PV/CO	Good tolerance	–	–
32	B	PV/CO	Good tolerance	–	–
33	A	PV/CX/CO	Good tolerance	–	–
34	B	PV	Good tolerance	–	–
		CO	Urticaria	25	50
35	B	CO	Good tolerance	–	–
36	A	PV/CX/CO	Good tolerance	–	–
37	B	PV/CO	Good tolerance	–	–
		CX	Urticaria	50	150
38	B	PV/CO	Good tolerance	–	–
39	B	PV/CX/CO	Good tolerance	–	–
40	A	PV/CX/CO	Good tolerance	–	-
41	A	PV/CX/CO	Good tolerance	–	-
42	A	PV/CX/CO	Good tolerance	–	–
43	A	PV/CX/CO	Good tolerance	–	–
44	A	PV/CX/CO	Good tolerance	–	–
45	A	PV/CX/CO	Good tolerance	–	–
46	A	PV/CX/CO	Good tolerance	–	–
47	A	CX/CO	Good tolerance	–	–
48	A	CX/CO	Good tolerance	–	–
49	A	PV/CX/CO	Good tolerance	–	–
50	A	PV/CX/CO	Good tolerance	–	–
51	A	PV/CX/CO	Good tolerance	–	–
52	A	PV/CX/CO	Good tolerance	–	–
53	B	PV/CO	Good tolerance	–	–
		CX	Anaphylaxis	30	50
54	A	PV/CX/CO	Good tolerance	–	–

Time Interval and Total cumulative dose drug administration and the development of symptoms

Pat, Patients; AX, Amoxicillin; PV, Penicillin V; CX, Cefadroxil; CO, Cefuroxime; IDR (min), Interval between drug administration and development of symptoms in minutes; TCD, Total cumulative dose in mg

P1, P5, P19, and P26 were diagnosed by DPT, with 2 cases developing urticaria, 1 anaphylaxis, and 1 generalised pruritus and erythema after AX administration (Table 3). P8 developed anaphylaxis after PV administration. Based on ST and DPT, patients were diagnosed as selective reactors to AX (N = 48, 88.9%) or allergic to the whole group of penicillins (N = 6, 11.6%).

In all cases, ST with CX was done, with 37 (68.5%) cases negative and 17 (31.5%) positive (6 (35.3) by SPT and 11 (64.7%) by IDT) (Table 2). From the 37 cases with negative ST to CX, DPT was done with this cephalosporin, being positive in 2 (5.4%) (Table 3). P37 developed urticaria in trunk 50 min after 150 mg of CX and needed antihistamines and corticosteroids, and P53 developed anaphylaxis 30 min after 50 mg of CX and needed epinephrine.

### Tolerance to cefadroxil happens in 65% of AX allergic patients

Based on CX study, 35 (64.8%) cases showed tolerance (Group A) and 19 (35.2%) cross-reactivity (Group B). Comparisons of the clinical characteristics of both groups showed no differences regarding type of the original reaction to penicillins, time interval between drug administration and symptom development, or time between last reaction and study. Comparisons of ST to AX showed that in Group A, 12 (34.3%) cases were positive by SPT, 19 (54.3%) by IDT and 4 (11.4%) negative whereas in Group B 15 (78.9%) cases were positive by SPT, 4 (21.1%) by IDT, and 0 (0%) negative, being differences statistically significant (p = 0.006).

The allergological study to CO showed negative STs in all cases (Table 2) and tolerance was also confirmed in all cases by DPT (Table 3) but only P34 (Group B) developed urticaria in trunk and arms 25 min after 50 mg of CO. Symptoms resolved 2 h after antihistamine administration. Therefore, cross-reactivity with CO was 1.8%, although a concomitant sensitisation rather than a cross-reactivity could be hypothesized.

### Significant differences of recognition are only found at the lower concentration of cefadroxil

The analysis of sIgE results indicated that the mean value of RAST to BPO-PLL and AXO-PLL was 1.12 ± 3.65 and 6.8 ± 9.4 respectively, with 4 out of 48 (8.3%) cases positive to BPO-PLL and 24 out of 48 (50%) to AXO-PLL (Table 2). Comparisons between groups A and B showed higher differences, although not discriminating, in terms of mean levels to AXO-PLL and the percentage of positive cases (76.5% vs 35.5%;) for AXO-PLL in Group B (p = 0.038 and p = 0.007, respectively).

**Fig. 4:**
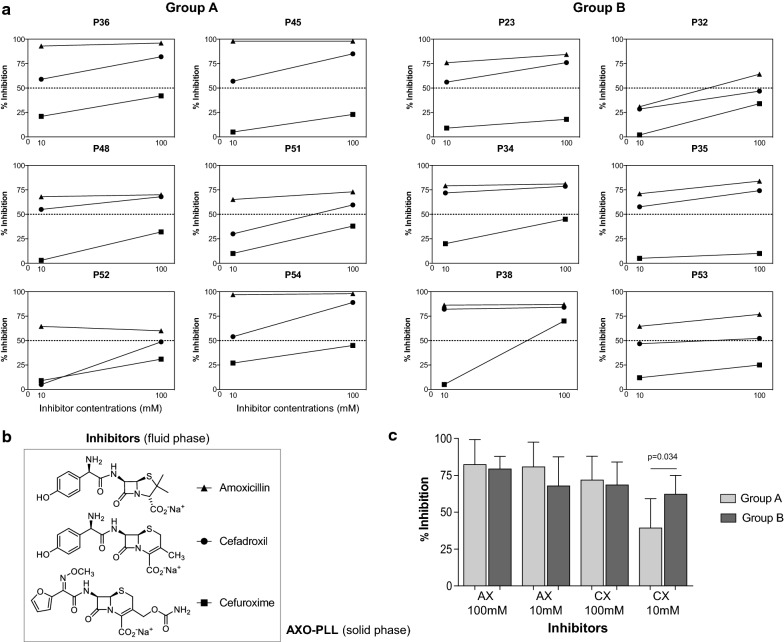
Immunological recognition of different BLs evaluated by RAST Inhibition using AXO-PLL as solid phase. **a** Graphs show the percentage of inhibition produced by different BLs, including amoxicillin, cefadroxil, and cefuroxime at two concentrations 10 and 100 mM in 6 individual sera from Group A (left) and 6 individual sera from group B (right). **b** Chemical structures of BLs, amoxicillin, cefadroxil and cefuroxime, used in the fluid phase. **c** Mean of the percentage of inhibition induced by Amoxicillin (AX) and Cefadroxil (CX) at two different concentrations in sera from AX-allergic patients from Groups A and B. Statistical analysis for non-related samples was performed by Mann–Whitney U test and significance considered for p < 0.05

To study CX specific recognition, we performed RAST inhibition assays on 6 cases from each group (Fig. 4a). As inhibitors, we included AX, CX, and CO at two concentrations, 10 and 100 mM (Fig. 4b). Results with AX showed, as expected, a high percentage of inhibition at both concentrations in all cases. Regarding CX, the percentage of inhibition was above 50% in most of patients at 100 mM, 5 out of 6 patients in each group, similarly to levels obtained with AX. However, these percentages decrease at 10 mM, being lower than those observed with AX especially Group A (Fig. 4a). In fact, comparison analysis of the percentage of inhibition between groups only shows significant differences for CX at 10 mM (p = 0.034) (Fig. 4c). Only one case (P38, Group B) showed a percentage above 50% with CO.

### Synthetic determinants of cefadroxil showed better sIgE recognition in Group B

The design of the two synthetic determinants of CX was based on our degradation hypothesis of the aminocephalosporin-protein conjugate, using butylamine as a model nucleophile emulating protein lysine (Fig. 3a). After covalent protein conjugation through BL ring, the dihydrothiazine ring is unstable and could degrade producing structures in which carbon 6 presents different functionalities. Two relevant candidates, according to previous immunological recognition results [30], are structures bearing hydroxyl and aldehyde functionality in carbon 6. In the case of hydroxyl functionality, it would generate the molecule **1** as determinant; whereas the aldehyde functionality can react with the amino group of R1 side chain generating the pyrazinone **2** as a novel determinant. The synthesis of the molecule **2** was achieved following the Ugi/Deprotect/Cyclize strategy (Fig. 3b) [39]. First, starting reagents (an isocyanide, a protected amine, a protected aldehyde, and a N-protected aminoacid) were assembled by following the one-pot Ugi four-component reaction to produce the Ugi adduct. The latter acid-mediated-cleavage of the protected groups may result in the amino-functionalised aldehyde intermediate that cyclises, through intramolecular imine formation, and aromatises affording target pyrazinone (**2**). This method allowed the straightforward synthesis of **2**, for which other procedures resulted unsuccessful. Compounds **1** and **2** were purified and well-characterised, allowing the immunological recognition study of precise chemical structures.

**Fig. 5:**
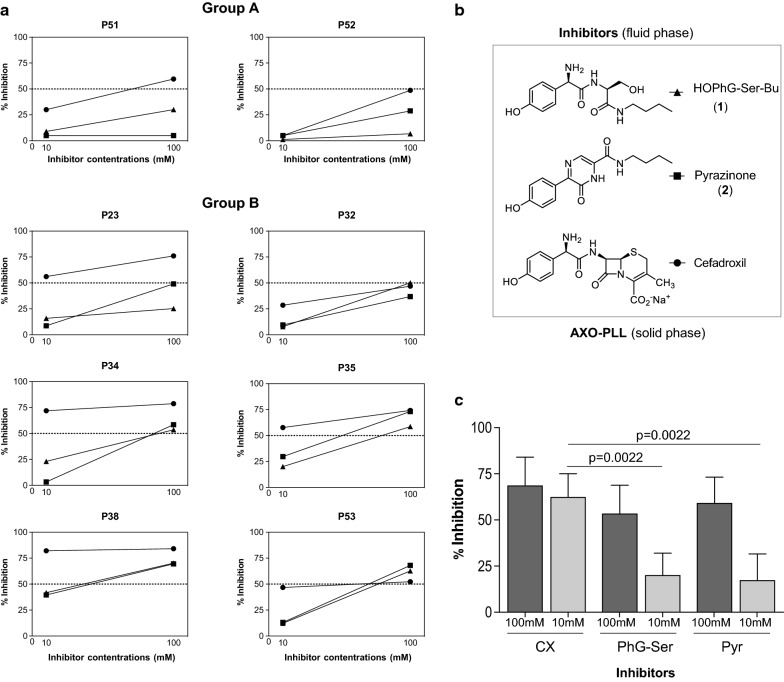
Immunological evaluation results with cefadroxil (CX) and its synthetic antigenic determinants. **a** RAST inhibition assays performed with sera from AX-allergic patients, 2 from Group A and 6 patients from Group B. **b** Chemical structure of inhibitors, synthetic compounds HOPhG-Ser-Bu (**1**), Pyrazinone (**2**) and native CX, and solid phase (amoxicilloyl-poly-L-Lysine, AXO-PLL). **c** Bars represent mean of percentage of inhibition for the three inhibitors at 100 and 10 mM concentrations in Group B. Statistical comparisons among related samples by Friedman test for related samples being significant with p values < 0.05

RAST inhibition assays were performed using CX and the two synthetic structures (**1** and **2**), as inhibitors (Fig. 5b), in two cases from Group A and 6 from Group B. There was no inhibition with these structures in Group A (Fig. 5a). Higher percentages of inhibition were observed in Group B, being greater than 50% in 4 out of 6 cases at 100 mM, in which similar levels of inhibition to those obtained with CX were observed (Fig. 5a). However, significant lower percentage of inhibition with these synthetic structures was observed performing the RAST inhibition at 10 mM (p = 0.0022 for both) (Fig. 5c).

## Discussion

BLs are the most widely used antibiotics and the drugs most frequently involved in IHR [1] in adults and children [40–42]. All BL compounds can potentially induce a specific immunological response and, due to their wide prescription, BL allergy is nowadays a worldwide health issue with relevant implications [43–45]. One of the main issues is establishing the risk of developing an allergic reaction to cephalosporins prescribed in patients previously diagnosed of penicillin IHR, with different unsolved questions like if this risk can be predicted by ST and/or DPT, or the role of the chemical structure, specifically the side chain, in this recognition [10, 46–49]. The main difficulty is that, despite efforts [28–30], the antigenic determinants of cephalosporins are unknown [31]. In this study we have found that, for predicting cross-reactivity, ST is not enough and, R1 side chain, although critical for recognition, is not the unique factor. Moreover, the use of chemical tools for SAR study is a promising approach for elucidating the chemical structures involved in these IHRs.

In this study we have found that the cross-reactivity of IHRs to AX with CX, a cephalosporin with the same R1, was 35% and with CO, cephalosporin with different R1, figures decrease to 1.8%. Results with CX are in agreement with those by Romano [19] reporting that 39.3% of patients with IHR to penicillins had positive tests for cephalosporins, with 37.7% positive to aminocephalosporins, including CX and/or cefamandole. These results are similar to our previous data, with 38% of cross-reactivity between AX and CX using DPT [21]. Relevantly, we did not detect differences in cross-reactivity to CX among those selective to AX compared to those reacting to the whole group, confirming that R1 is not the only factor influencing cross-reactivity. Regarding CO tolerance, all patients had negative ST and only one had a positive DPT, showing a high degree of CO tolerance, in line with previous data [16–19, 25].

Comparisons of SPT results to AX between Group A and B (cross-reactivity to CX) showed a higher percentage of positivity (78.9 vs 34.3%) in the group tolerant to CX (Group A). These results agree with those by Romano [19] estimating an odds ratio of ST positivity to ampicillin for cross-reacting to at least one cephalosporin of 2.5 (CI, 1.4–4.5). Moreover, the analysis of the sIgE results showed significantly higher levels and positivity to AXO-PLL in Group B. This seems to indicate that patients that cross-react with cephalosporins have a high degree of reactivity, taking into account that the two cases that developed systemic symptoms after ST with penicillins belong to Group B and that patients reacted to small amount of CX (50 and 150 mg) and CO (50 mg) in DPT.

Regarding the role of ST for predicting cross-reactivity, a positive cephalosporin ST in patients allergic to penicillins may indicate not only cross-reactivity but also concomitant sensitivity. Of note, P34 with cross-reaction to CO also reacted to CX. Whether this patient has cross-reacting or co-existing antibodies was something we cannot clarify in the present study as the RAST level was not enough for performing RAST inhibition with both drugs. However, cross-reactivity is more probable since this patient had not been previously treated with cefuroxime or any cephalosporin. This percentage is in agreement with previous data [18] that found 2.9% of cross-reactivity with CO in patients with prior histories involving only a penicillin. If a negative cephalosporin ST predicts good tolerance is controversial [50]. Different studies showed that patients with a well-established IgE-mediated allergy to penicillin and with ST negative to cephalosporins tolerate cephalosporins [15–17]. However, others demonstrated that less than 3% of cases can have a DPT positive with cephalosporin despite having negative ST [18, 19]. In this study 2 out of 37 patients (5.4%) with ST negative to CX and 1 out of 54 patients (1.8%) with ST negative to CO had a positive DPT to CX and CO respectively, indicating a negative predictive value (NPV) of 94.6% for CX and 98.1% for CO. That means that although NPV are high, a negative ST does not mean tolerance even if R1 are different.

Our immunological study by RAST inhibition assays agrees with previous results on cross-reactivity between penicillins and cephalosporins, showing that AX presented a better recognition, followed by CX [21]. Data showed a discriminating capacity of the test between Group A and B using lower drug concentrations, 10 mM, observing a significantly lower recognition of CX in patients with good tolerance to CX (Group A).

Regardless of this discriminative capacity, these data indicate that, although important for IgE recognition, the R1 is not per se the only structure involved in the immunological response, as structural modifications or some fragments of the nuclear structure may be involved in the antigenic determinant. In penicillins, the penicilloyl structure formed after protein conjugation is stable and, therefore, the thiazolidine ring could also play a role in the antigenic determinant [51–53]. On the contrary, the equivalent cephalosporyl structure is unstable, thus the R2 substituent is expulsed [54, 55] and the dihydrothiazine ring suffers different fragmentations, producing a complex mixture in which structures are difficult to elucidate [29, 31]. We have addressed this issue, by using chemical tools, for performing SAR studies in which precisely defined structures, consisting on the R1 side chain coupled to the open BL ring with the carbon 6 of the original drug represented by a methyl group, were recognised by sIgE from patients with IHR to the cephalosporin containing either the same R1 or one structurally similar [29]. Further SAR studies involved similar synthetic determinants but with different functionalisation in such carbon 6, finding that hydroxymethyl and aldehyde functionality, compared with methyl group, increased recognition [30]. Based on these results, synthetic determinants of CX, involving the whole intact R1 or a modified R1 side chain, have been immunologically evaluated, showing higher-recognition by sIgE from patients cross-reactive to CX (Group B).

The structure **1** (HPhG-Ser-Bu), consisting on the R1 side chain of CX and open BL ring with hydroxymethyl functionality at carbon 6 [30], was not previously evaluated with sIgE to aminocephalosporins. These determinants containing the intact corresponding aminocephalosporins R1 have been immunologically evaluated in a recent study with cefaclor-allergic patients (12% of positive cases) [28], and in the present study with AX- and/or CX-allergic patients (66% of positive cases at the maximum concentration), showing different extent of recognition depending on R1.

The pyrazinone **2** has been synthesised and immunologically evaluated in this study for the first time. Its structure derives from intramolecular reaction between the R1 amino group and the aldehyde at carbon 6. Inhibition results in six cases of Group B show that the pyrazinone **2,** at 100 mM concentration, is recognised in 66% of cases, in agreement with IgE recognition observed for pyrazinones derived from cefaclor, with 63% of positive cases for the equivalent pyrazinone to that described here [28], and 60% of patients for an equivalent analog developed by Venemalm [32].

These synthetic determinants (**1** and **2**) were not recognised by the two selected patients with tolerance to CX (Group A). Importantly, greater differences in recognition between CX and the synthetic structures were observed in Group A than in Group B, using the higher concentration.

One could think that AX presents the amino group in R1 for the formation of additional determinants, as diketopiperazine, considered as a minor determinant of AX [56]. However, it did not show sIgE recognition in previous studies [57], which is consistent with its lack of reactivity with proteins [56].

## Conclusions

We have confirmed that cross-reactivity between penicillin and cephalosporins occurs when the R1 side chain is identical as previously reported, and that negative ST is not enough for predicting tolerance, being DPT necessary. The primary determinant of immunochemical recognition of aminocephalosporins rested, with the structure of the R1, intact (molecule **1**) or in its cyclised form as pyrazinone (molecule **2**), although other parts of the molecule (excluding R2 substituents and most of the dihydrothiazine) are necessary for the formation of the antigenic determinant. These structures represent useful and safe alternatives for determining in vitro cross-reactivity to CX in AX-allergic patients. We think that other determinants, involving different patterns of recognition, could also participate in CX-allergic reactions; and more research is needed in this regard.

## Supplementary Information

**Additional file 1: Figure S1.** Nuclear Magenteic Resonance (NMR) characterization of structure 2. (A) ^1^H-NMR (CH_3_OD)spectrum, (B) ^13^C-NMR (CH_3_OD) spectrum, and (C) heteronuclear single quantum coherence (HSQC) experiment with gradient pulse. Bidimensional NMR spectrum (left) and signal assignation (right).

## Data Availability

All data generated or analysed during this study are included in this published article and its supplementary Additional file 1.

## Abbreviations

AX: Amoxicillin
AXO: Amoxicilloyl
BL: Betalactam
BP: Benzylpenicillin
BPO: Benzylpenicilloyl
BP-OL: Benzylpenicilloyl-octa-L-lysine
CO: Cefuroxime
CX: Cefadroxil
DPT: Drug provocation test
EAACI: European Academy of Allergy and Clinical Immunology
IDT: Intradermal tests
IHR: Immediate hypersensitivity reactions
IR: Interquartile range
MD: Minor determinant
MDM: Minor determinant mixture
NPV: Negative predictive value
PPL: Benzylpenicilloyl-poly-L-lysine
PLL: Poly-L-Lysine
PV: Penicillin V
RAST: Radioallergosorbent test
SAR: Structure–activity relationship
sIgE: Specific IgE
ST: Skin testing
SD: Standard deviation
SPT: Skin prick test
TCD: Total cumulative dose
