# Supplementary material for: Penicillin and cephalosporin cross-reactivity: role of side chain and synthetic cefadroxil epitopes
Source: Clin Transl Allergy. 2020 Dec 4;10:57. doi: 10.1186/s13601-020-00368-1 (PMC7716594; doi:10.1186/s13601-020-00368-1)
Supplement: Supplementary file 1 — Additional file 1: Figure S1. Nuclear Magenteic Resonance (NMR) characterization of structure 2. (A) 1H-NMR (CH3OD)spectrum, (B) 13C-NMR (CH3OD) spectrum, and (C) heteronuclear single quantum coherence (HSQC) experiment with gradient pulse. Bidimensional NMR spectrum (left) and signal assignation (right). [file 13601_2020_368_MOESM1_ESM.pdf]

Additional File 1: ***Synthesis and characterization of cefadroxil determinants***

Penicillin and cephalosporin cross-reactivity: Role of side chain and synthetic cefadroxil epitopes.

Gador Bogas, MD, PhD[1,2*], Cristobalina Mayorga, PhD[1,2,3*], Ángela Martín-Serrano, PhD[1,3], Rubén Fernández-Santamaría, BSc[1], Isabel María Jiménez-Sánchez, BSc[1,3], Adriana Ariza, PhD[1], Esther Barrionuevo, MD, PhD[1,2], Teresa Posadas, MD, PhD[1,2], María Salas, MD, PhD[1,2], Tahía Diana Fernández, PhD[1,2], María José Torres, MD, PhD[1,2,3,4#], María Isabel Montañez, PhD[1,3#]

* or # authors with the same contribution

[1]Allergy Research Group, Instituto de Investigación Biomédica de Málaga-IBIMA. Hospital Civil, 29009 Málaga, Spain

[2]Allergy Unit, Hospital Regional Universitario de Málaga. Hospital Civil, 29009 Málaga, Spain

[3]Nanostructures for Diagnosing and Treatment of Allergic Diseases Laboratory, Andalusian Center for Nanomedicine and Biotechnology-BIONAND. Parque Tecnológico de Andalucía, 29590 Málaga, Spain

[4]Departamento de Medicina, Universidad de Málaga. Facultad de Medicina, 29071 Málaga, Spain

Corresponding author

Maria Jose Torres Jaen , MD, PhD

Allergy Service, Hospital Civil

Plaza Hospital Civil s/n 29009 Malaga, Spain

e-mail: mjtorresj@ibima.eu

Table of contents

Synthesis of chemical structures……………………………………………………………….2

Synthetic determinant **1**…………………………………………………………………….2

Synthetic determinant **2**…………………………………………………………………….2

Figure S1. Nuclear Magneticic Resonance (NMR) characterization of structure **2**……………...3

References…………………………………………………………………………………….4

Synthesis of chemical structures

Synthetic determinant 1 (HOPhG-SerBu) named ((2S)-N^2-{[(2R)-amino (4hydroxyphenyl) acetyl]amino}-N^1-butylserinamide hydrochloride)).

This was synthesized following six synthetic steps using serine, N-butylamine and hydroxyphenylglycine as starting reagents, as previously described.[1]

Synthetic determinant 2 (pyrazinone) named (N-butyl-5-(4-hydroxyphenyl)-6-oxo-1,6-dihydropyrazine-2-carboxamide)). It was synthesized following the Ugi/Desprotect/Cyclize (UDC) strategy (Figure 3B),[2] adapting protocols from cefaclor pyrazinone synthesis.[3] Equimolar amounts of each reagent (butyl isocyanide, 2,5-dimethoxybenzylamine, 2,2-dimethoxyacetaldehyde solution 60% in H_2O and *N*-Boc-hydroxyphenylglycine) were left to react in methanol at room temperature for 48 hours, until Ugi adduct formation was complete (thin layer chromatography evidence). To remove possible remaining isocyanide, dichloromethane and polystyrene-supported p-toluenesulfonic acid were added and the mixture was stirred at room temperature for 90 minutes. The polystyrene-supported p-toluenesulfonic acid was filtered off and successively washed with methanol, ethyl acetate and dichloromethane (three times). To deprotect and cyclize, the residue obtained after solvent evaporation (Ugi adduct) was treated with 30% trifluoroacetic acid in dichloroethane and heated at 80˚C for 2 hours. The solution was then treated with a saturated solution of $NaHCO_3$ until pH 8 and extracted with ethyl acetate. The aqueous phase was treated with a saturated solution of $NaHCO_3$ until pH 7 and extracted again with ethyl acetate. Then, organic phases were combined, dried over $MgSO_4$ and evaporated under reduced pressure to produce an oily yellow residue that was purified by chromatographic methods on silica gel using dichloromethane/methanol mixtures, with the addition of 2% NH_3 to obtain high recovery. Bright (dark) yellow solid, 25% yield. Melting point = 219 - 222 ºC. $[\alpha]_D$= - 49 (c_1 = 0.2, methanol). ^{1}H-NMR (400 MHz, MeOD): δ (ppm) 8.39 (s, 1H; N-CH=CCO), 8.17 (d, ^3J(H,H) = 9.5 Hz, 2H; Aromatic-ortho), 6.86 (d, ^3J(H,H) = 9.5 Hz, 2H; Aromatic-meta),

3.39 (t, 2H; -CONH-C$\underline{H}_2$-), 1.60 (m, 2H; -CH$_2$-C$\underline{H}_2$-CH$_2$-), 1.40 (m, 2H; -CH$_2$-C$\underline{H}_2$-CH$_3$), 0.97 (t, ^3J(H,H) = 7.6 Hz, 3H; -CH$_2$-C$\underline{H}_3$). ^{13}C-NMR (150 MHz, MeOD): δ (ppm) 164.1, 160.9, 157.1, 150.5, 135.6, 131.7, 130.4, 128.0, 115.9, 40.4, 32.6, 21.1, 14.1. Electrospray ionization-mass spectrometry (ESI-MS) calculated for $C_{15}H_{17}N_3O_3$ $(M + H)^+$: 288.1348, found: 288.1342.

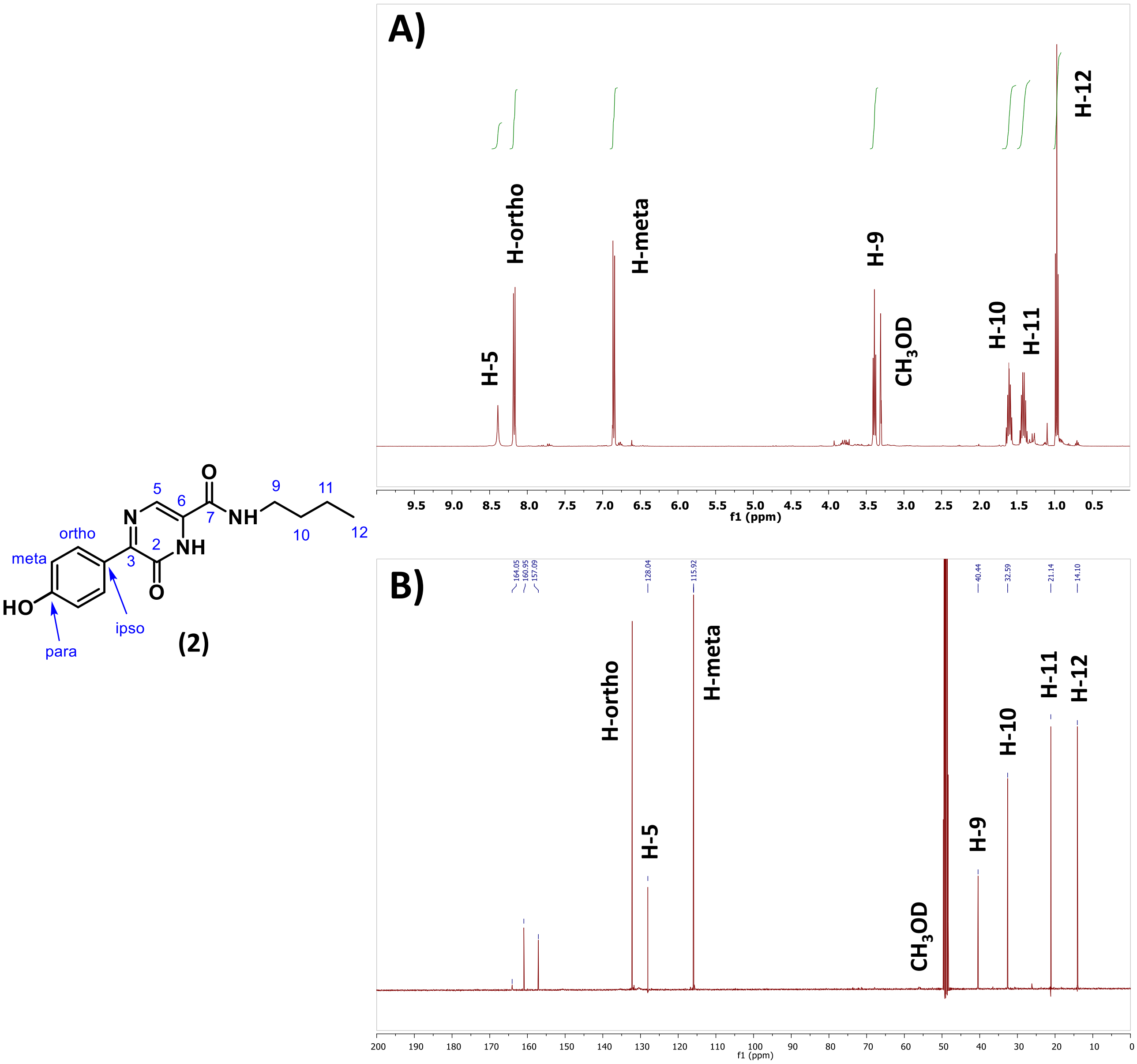

C)

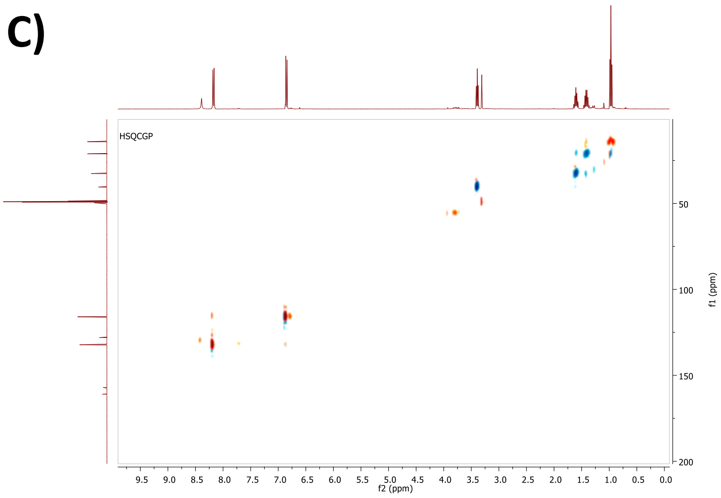

position	^{1}H-NMR (CH_3OD) δ (ppm)	^{13}C-NMR (CH_3OD) δ (ppm)
5	8.40 (s, 1H)	128.1
Ortho	8.20 (d, 2H, *J* = 9.5 Hz)	132.1
Meta	6.86 (d, 2H, *J* = 9.5 Hz)	115.9
9	3.39 (t, 3H, *J* = 7.2 Hz)	40.4
10	1.61 (m, 2H)	32.6
11	1.43 (m, 2H)	21.1
12	0.99 (t, 3H, *J* = 7.6 Hz)	14.1

Figure S1. Nuclear Magenteic Resonance (NMR) characterization of structure **2**. (A) ^{1}H-NMR (CH_3OD) spectrum, (B) ^{13}C-NMR (CH_3OD) spectrum, and (C) heteronuclear single quantum coherence (HSQC) experiment with gradient pulse. Bidimensional NMR spectrum (left) and signal assignation (right).

References:

1. Montañez MI, Mayorga C, Torres MJ, Ariza A, Blanca M, Perez-Inestrosa E. Synthetic Approach to Gain Insight into Antigenic Determinants of Cephalosporins: In Vitro Studies of Chemical Structure−IgE Molecular Recognition Relationships. Chemical Research in Toxicology 2011; 24:706-17.
2. Azuaje J, El Maatougui A, Pérez-Rubio JM, Coelho A, Fernández F, Sotelo E. Multicomponent Assembly of Diverse Pyrazin-2(1H)-one Chemotypes. The Journal of Organic Chemistry 2013; 78:4402-9.
3. Martín-Serrano A, Mayorga C, Barrionuevo E, Pérez N, Romano A, Moreno E, et al. Design of an antigenic determinant of cefaclor: Chemical structure–IgE recognition relationship. Journal of Allergy and Clinical Immunology 2020; 145:1301-4.e4.
